# ATPase Inhibitory Factor 1 Drives Mitochondrial Energy Metabolic Reprogramming to Promote HCC Vasculogenic Mimicry via the ESR1/miR-20a-3p/GNAZ Pathway

**DOI:** 10.34133/research.0998

**Published:** 2025-11-25

**Authors:** Shilun Wu, Changyu Yao, Lu Fang, Yiwen Sun, Mingming Zhao, Jie Chen, Gaofei Hu, Zhe Zhao, Shusi Ding, Jing Xue, Xiaoyi Liu, Wenbing Sun, Jian Kong, Lemin Zheng

**Affiliations:** ^1^ Department of Hepatobiliary Surgery, Beijing Chaoyang Hospital Affiliated to Capital Medical University, Beijing 100043, China.; ^2^ The Institute of Cardiovascular Sciences and Institute of Systems Biomedicine, School of Basic Medical Sciences, State Key Laboratory of Vascular Homeostasis and Remodeling, NHC Key Laboratory of Cardiovascular Molecular Biology and Regulatory Peptides, Beijing Key Laboratory of Cardiovascular Receptors Research, Health Science Center, Peking University, Beijing 100191, China.; ^3^ Research Center for Cardiopulmonary Rehabilitation, University of Health and Rehabilitation Sciences Qingdao Hospital (Qingdao Municipal Hospital), School of Health and Life Sciences, University of Health and Rehabilitation Sciences, Qingdao 266071, China.; ^4^ Department of Pathology, Peking University People’s Hospital, Peking University, Beijing 100044, China.; ^5^ Department of Cardiology and Institute of Vascular Medicine, Peking University Third Hospital, Beijing, China.; ^6^ Department of Pharmacy, Peking University Third Hospital; Institute for Drug Evaluation, Peking University Health Science Center, Beijing 100191, China.; ^7^ Beijing Tiantan Hospital, China National Clinical Research Center for Neurological Diseases, Advanced Innovation Center for Human Brain Protection, Beijing Institute of Brain Disorders, The Capital Medical University, Beijing 100050, China.

## Abstract

Vasculogenic mimicry (VM) is a microcirculation pattern that has a crucial effect on hepatocellular carcinoma (HCC) metastasis. In this study, leveraging the GeneCard and The Cancer Genome Atlas databases, we identified ATPase inhibitory factor 1 (IF1) as a potential regulator of VM formation. Our research findings indicate that IF1 can promote HCC cell tube formation in vitro and enhance HCC VM and lung metastasis in vivo. Transcriptome sequencing combined with in vivo experiments revealed that IF1 knockdown elevates miR-20a-3p expression. Lentivirus-mediated miR-20a-3p overexpression reversed IF1-induced VM. Dual-luciferase reporter gene assays showed that estrogen receptor 1 (ESR1) acts as a transcription factor of the miR-20a-3p precursor. Further mechanistic studies revealed that excessive reactive oxygen species accumulation caused by IF1-induced mitochondrial metabolic reprogramming can inhibit ESR1 expression by promoting DNA methylation of its promoter. G protein subunit alpha Z (GNAZ), a miR-20a-3p target protein, can promote VM by phosphorylating components of the ERK pathway. Collectively, these results delineate a novel IF1/ESR1/miR-20a-3p/GNAZ axis in HCC VM and metastasis, providing potential therapeutic targets.

## Introduction

Liver cancer is the third highest cause of death associated with cancer globally, with hepatocellular carcinoma (HCC) representing nearly 90% of primary liver cancer instances [1]. Although innovative treatment methods for HCC have been developed, patient survival rates remain unsatisfactory, largely because of tumor recurrence, metastasis, and treatment resistance [2,3]. As a hypervascular solid tumor, HCC exhibits the typical feature of abnormal angiogenesis that is essential for tumor invasion and metastasis. Using this model, various antiangiogenic therapies are commonly employed in clinical treatment, but their effectiveness is not at an acceptable level [4].

Vasculogenic mimicry (VM) is a novel concept that has gained traction in the field of cancer biology [5]. Unlike the well-established process of angiogenesis, which involves the growth of new blood vessels from preexisting ones, VM describes a unique mechanism wherein aggressive cancer cells can spontaneously organize themselves into structures that resemble blood vessels [6]. This phenomenon enables these malignant cells to secure direct access to vital nutrients and oxygen, circumventing the reliance on the body’s traditional vascular network. Various molecular pathways and elements play a role in the formation of VM, primarily including the epithelial–mesenchymal transition (EMT), hypoxic tumor microenvironment (TME), and extracellular matrix (ECM) remodeling [7–9]. Growing evidence indicates that VM is considerably linked to the malignant characteristics and unfavorable outcomes of HCC, representing a potential challenge for classical antiangiogenic treatment methods for this disease [10]. Thus, the biological features and underlying mechanisms related to VM, a critical anti-HCC therapeutic target, urgently need further investigation.

In mitochondria, adenosine triphosphate (ATP) synthase catalyzes ATP synthesis using the respiratory chain-generated proton electrochemical gradient as a driving force [11]. ATPase inhibitory factor 1 (IF1) is a small nuclear-encoded mitochondrial protein that is involved in regulating energy metabolism by directly binding with the βF1 subunit of ATP synthase [12]. IF1 expression levels are greatly increased in a number of human cancers, including HCC. Research has suggested that IF1 up-regulation can inhibit oxidative phosphorylation (OXPHOS) activity and induce a metabolic switch to enhanced aerobic glycolysis, which supports an oncogenic role of IF1 [13,14].

Based on the established role of IF1 in promoting HCC progression through mechanisms like EMT and angiogenesis [15,16], and considering the critical contribution of EMT to VM formation [7], we hypothesized that IF1 might also play a important role in VM within HCC, as suggested by GeneCard and The Cancer Genome Atlas (TCGA) databases. However, the specific impact of IF1 on VM and the underlying molecular mechanisms remain largely unexplored. Therefore, this study aimed to investigate the involvement of IF1 in HCC VM formation and elucidate the regulatory pathways involved.

Our findings demonstrate that IF1 indeed promotes VM in vitro and in vivo. Mechanistically, we identified that IF1, through inducing mitochondrial metabolic reprogramming and reactive oxygen species (ROS) accumulation, suppresses estrogen receptor 1 (ESR1) expression via promoter DNA methylation. ESR1, acting as a transcription factor for miR-20a-3p, consequently leads to decreased miR-20a-3p levels. This down-regulation releases its inhibition on G protein subunit alpha Z (GNAZ), which, in turn, promotes VM by activating the ERK/c-Fos/c-Jun signaling axis. Collectively, our results delineate a novel IF1/ESR1/miR-20a-3p/GNAZ pathway regulating VM in HCC, highlighting IF1 as a potential therapeutic target for suppressing VM-mediated metastasis.

## Results

### IF1 expression is associated with VM formation and unfavorable HCC patient prognostic outcomes

**Fig. 1.**
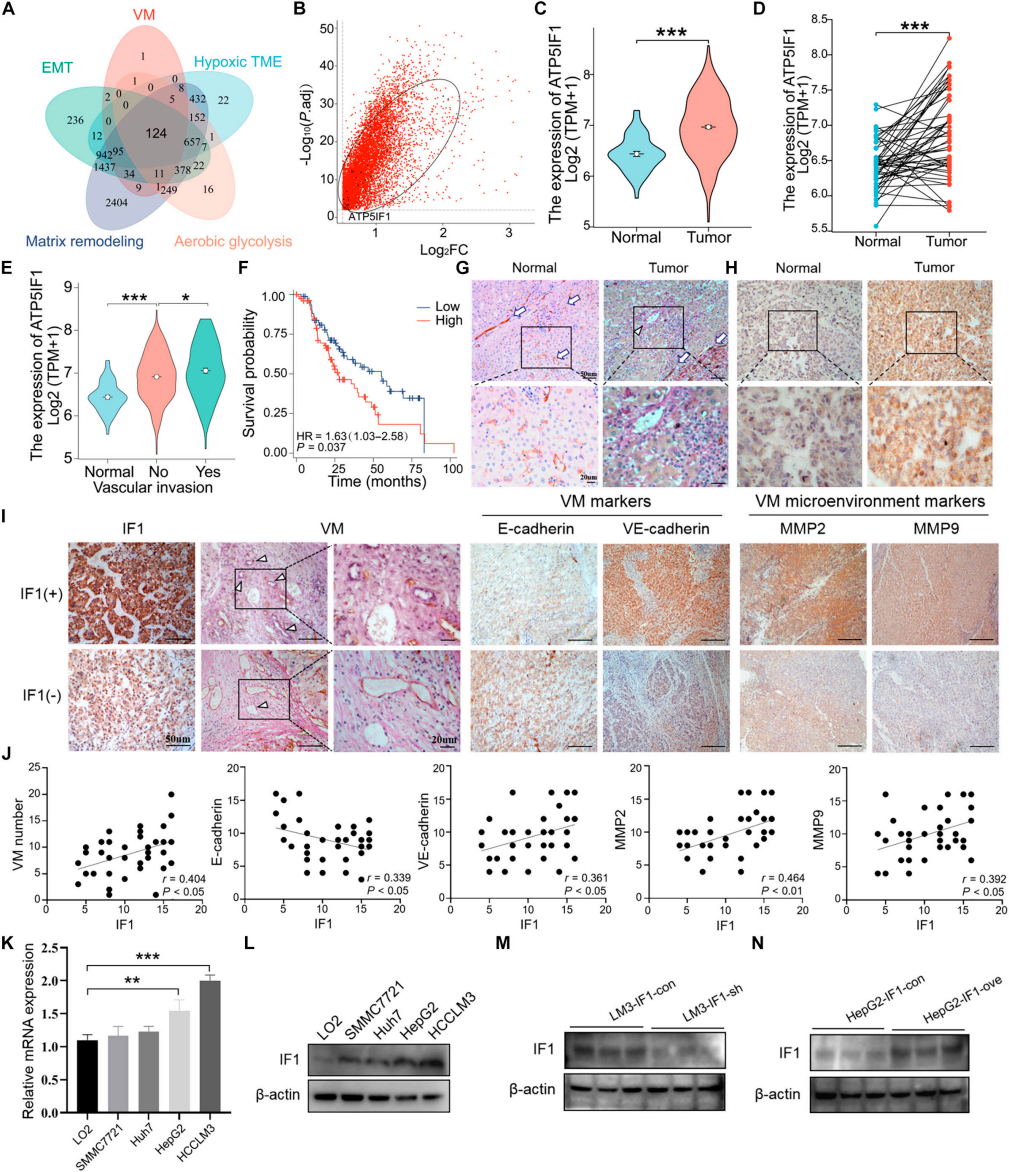
IF1 expression is associated with VM formation and poor prognosis in patients with HCC. (A) Venn diagram of VM-related genes. (B) Intersection of VM-related genes and differentially expressed genes from TCGA. (C and D) The TCGA database showed IF1 expression levels in HCC and normal tissues. (E) The TCGA database showed IF1 expression levels in HCC with and without vascular invasion. (F) Kaplan–Meier plot of overall survival of patients with HCC stratified by IF1 expression. (G) Representative images showing typical malignant morphology of vasculogenic mimicry by CD31 (brown)/PAS (pink)/hematoxylin (blue) staining in HCC. PAS+/CD31+, blood vessels; PAS+/CD31−, VM vessels. (H) Immunohistochemistry analysis showed IF1 expression levels in HCC and normal tissues. (I) IHC staining of VM, E-cadherin, VE-cadherin, MMP2, and MMP9 in IF1-high and IF1-low HCC tissue samples. (J) Statistical analysis of the correlation analysis between IF1 and VM density, E-cadherin, VE-cadherin, MMP2, and MMP9. (K) Relative expression level of IF1 in normal hepatocyte lines (LO2) and HCC cell lines (SMMC7721, Huh7, HepG2, and HCCLM3) by RT-qPCR analysis. (L) Relative expression level of IF1 in normal hepatocyte lines (LO2) and HCC cell lines (SMMC7721, Huh7, HepG2, and HCCLM3) by Western blot analysis. (M) The IF1 expression level in control and IF1-knockdown HCCLM3 cells. (N) The IF1 expression level in control and IF1-overexpressing HepG2 cells. Error bars represent mean ± SD. The *t* test was used for statistical analysis (ns, not significant; *P < 0.05, **P < 0.01, ***P < 0.001).

Previous studies have shown that aerobic glycolysis, hypoxic TME, EMT, and ECM remodeling are all involved in the VM formation; we obtained a total of 291 VM-related genes (Table S1), 1,624 aerobic glycolysis-related genes (Table S2), 2,457 hypoxic TME-related genes (Table S3), 3,957 EMT-related genes (Table S4), and 6,938 ECM remodeling-related genes (Table S5) from GeneCard. After taking the intersection of the Venn diagram, 124 genes were identified (Fig. 1A). Then, we obtained the RNA data of 158 HCC and 50 normal tissue samples from the TCGA database. By intersecting the 105 genes with differentially expressed genes (DEGs) from the TCGA database, we further narrowed down the selection scope (Fig. 1B). Through bioinformatics data analysis, in those genes, we found that IF1 expression levels were significantly higher in HCC than in peripheral normal tissues (Fig. 1C and D). Significantly, in HCC with vascular invasion, the expression of IF1 is significantly elevated (Fig. 1E). Furthermore, higher IF1 expression patterns were associated with poor patient prognosis (Fig. 1F). Hence, IF1 was selected for subsequent research.

First, to examine VM formation in liver tissues, CD31 and Periodic acid–Schiff (PAS) dual staining was applied and VM was detected only in HCC tissues and not in normal hepatic tissues (Fig. 1G). Through immunohistochemistry (IHC) staining, we confirmed that IF1 expression was increased in HCC tissues (Fig. 1H). Next, we first investigated the correlation between IF1 expression and VM density, with the results revealing a positive correlation between IF1 expression levels and VM density in HCC tissues (Fig. 1I and J). Then, we examined VM and VM microenvironment-associated markers in HCC tissues by IHC analysis and observed that IF1 expression levels were inversely related to expression levels of E-cadherin and positively related to those of matrix metalloproteinase-9 (MMP9), matrix metalloproteinase-2 (MMP2), and VE-cadherin (Fig. 1I and J). These results collectively demonstrated that IF1 participates in VM formation and is closely related to poor prognosis in HCC. Then, we examined IF1 expression levels in healthy hepatic cells (LO2) and different HCC cell lines (HepG2, Huh7, SMMC7721, and HCCLM3). IF1 expression levels were significantly increased in HepG2 and HCCLM3 cells compared with LO2 cells (Fig. 1K and L), with higher levels in HCCLM3 cells relative to HepG2 cells. To further analyze the mechanism underlying its role in VM, IF1 was knocked down in HCCLM3 cells (Fig. 1M and Fig. S1A) and overexpressed in HepG2 cells (Fig. 1N and Fig. S1B).

### IF1 promotes HCC VM formation and tumor growth

**Fig. 2.**
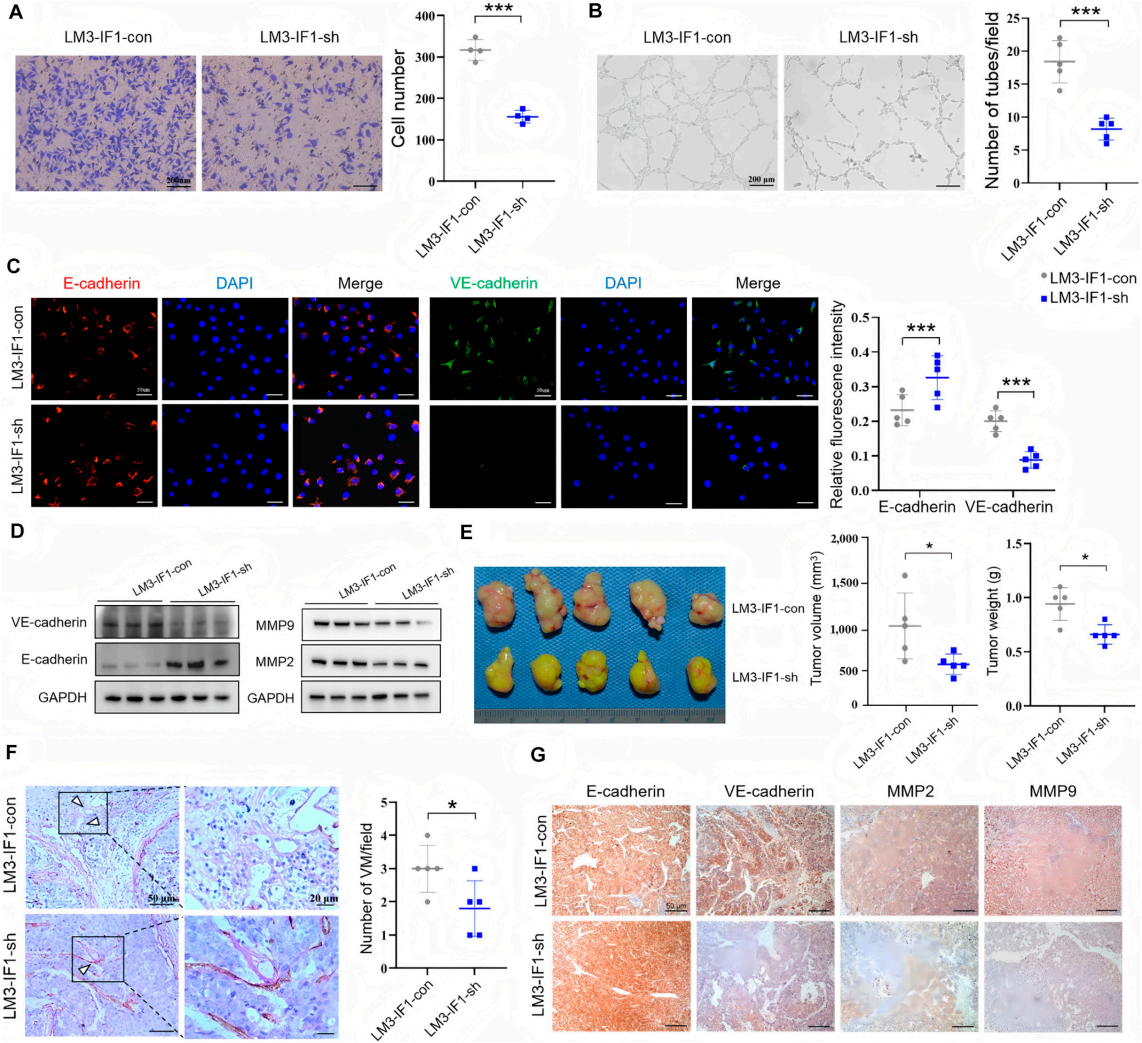
IF1 promotes HCC VM formation and tumor growth. (A) Transwell migration assay showing significantly reduced migration ability of HCCLM3 cells after IF1 knockdown. (B) Tube formation assay showing significantly decreased number of tube-like structures formed by HCCLM3 cells after IF1 knockdown. (C) Immunofluorescence staining showing increased E-cadherin expression and decreased VE-cadherin expression in IF1-knockdown HCCLM3 cells. (D) Western blot showing up-regulated E-cadherin protein expression and down-regulated VE-cadherin, MMP2, and MMP9 protein expression in IF1-knockdown HCCLM3 cells. (E) Nude mouse subcutaneous xenograft model showing that mice injected with IF1-knockdown HCCLM3 cells had significantly smaller tumor volume and lighter tumor weight than the control group (*n* = 5/group). (F) CD31/PAS staining showing significantly lower VM density in tumor tissues of the IF1-knockdown group than in the control group. (G) IHC staining showing down-regulated VE-cadherin, MMP2, and MMP9 expression and up-regulated E-cadherin expression in tumor tissues of the IF1-knockdown group. Error bars represent mean ± SD. The *t* test was used for statistical analysis (ns, not significant; *P < 0.05, ***P < 0.001).

Next, we explored the effect of IF1 on HCC VM through in vivo and in vitro experiments. IF1 knockdown significantly decreased the invasion ability of HCCLM3 cells (Fig. 2A) and inhibited their tube formation (Fig. 2B), as assessed by Transwell and tube formation assays, respectively. Then, we investigated the effects of IF1 on VM-associated marker expression patterns. Immunofluorescence results showed high expression of E-cadherin and low expression of VE-cadherin in IF1-knockdown HCCLM3 cells (Fig. 2C). Western blot analysis results further suggested that IF1 knockdown could up-regulate E-cadherin protein expression levels and down-regulate VE-cadherin, MMP-2, and MMP-9 protein expression levels in HCCLM3 cells (Fig. 2D and Fig. S1C). We then investigated if IF1 could promote HCC VM in vivo. We established a nude mouse subcutaneous xenograft tumor model and found that the tumor volume and weight were decreased in mice subcutaneously injected with IF1-knockdown HCCLM3 cells compared with the control group (Fig. 2E). We also found that IF1 knockdown could significantly inhibit VM formation in tumors (Fig. 2F). Moreover, IHC analysis revealed that the VE-cadherin, MMP2, and MMP9 protein expression levels were significantly down-regulated, while those of E-cadherin were up-regulated, in IF1-knockdown HCCLM3 tumors compared with control tumors (Fig. 2G). IF-overexpressing HepG2 cells showed the opposite trends. IF1 overexpression enhanced HCC cell migration (Fig. S1D) and tube formation (Fig. S1E and F). Immunofluorescence assays also showed the opposite patterns compared with IF1-knockdown HCCLM3 cells (Fig. S1G). Western blot analysis revealed down-regulated E-cadherin protein expression levels, while markedly up-regulated VE-cadherin, MMP2, and MMP9 protein expression levels were observed in IF1-overexpression HepG2 cells (Fig. S1H). Moreover, the mice injected with the IF1-overexpression HepG2 cells showed higher tumor volume (Fig. S1I) and VM density (Fig. S1J) than the controls. IHC analysis of tumor tissues showed similar results to the Western blot analysis of IF1-overexpression HepG2 cells (Fig. S1K). Taken together, our data suggest that IF1 is important for promoting HCC VM formation and tumor growth.

### RNA transcriptome sequencing reveals that IF1 can inhibit miR-20a-3p expression

**Fig. 3.**
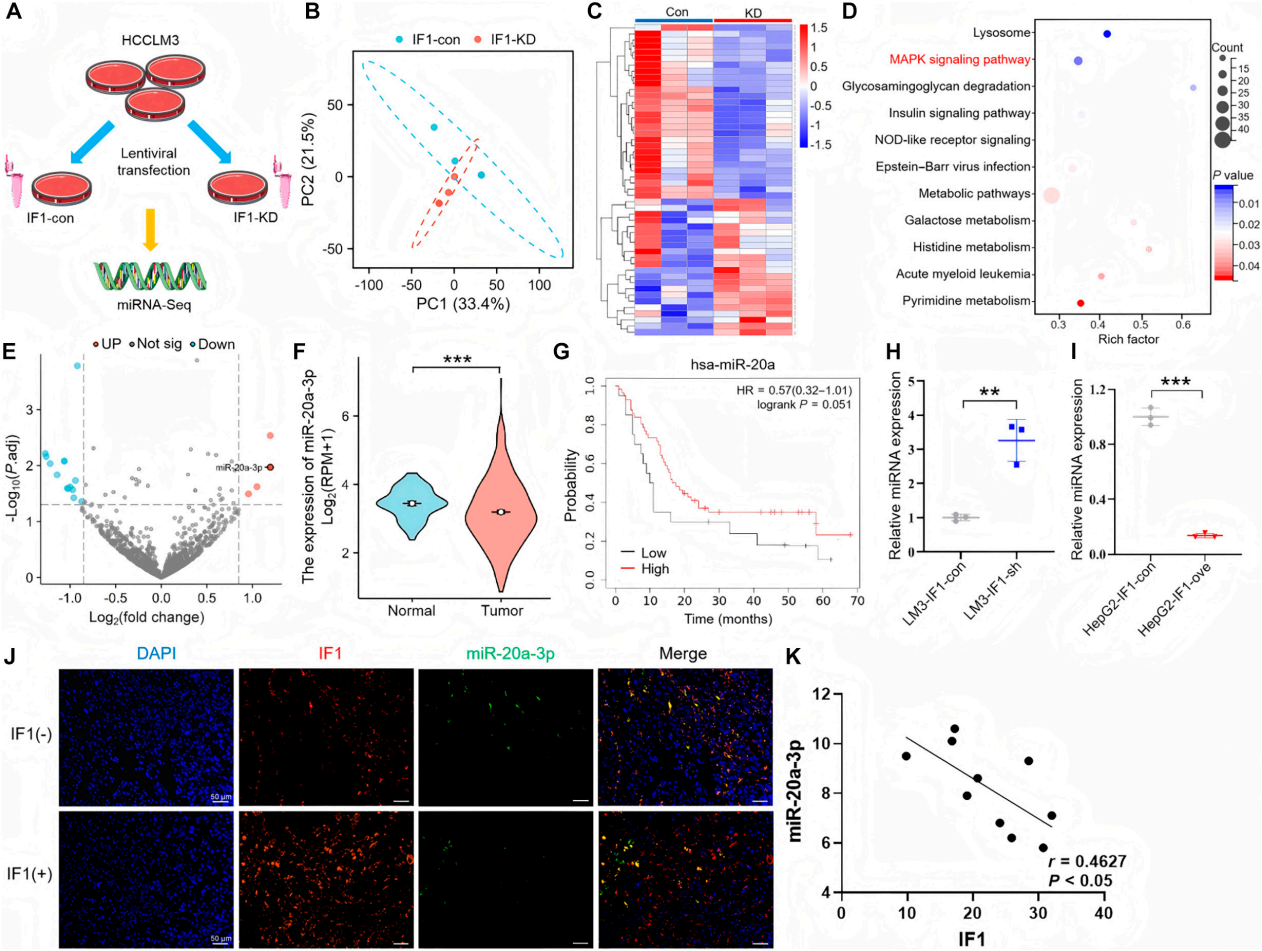
RNA transcriptome sequencing reveals that IF1 inhibits the expression of miR-20a-3p. (A) Schematic of the experimental workflow: miRNA-seq was performed on IF1-knockdown and control HCCLM3 cells. (B) PCA showing distinct separation of miRNA expression profiles between IF1-knockdown and control cells. (C) Cluster heatmap showing the distribution pattern of differentially expressed miRNAs between the 2 groups, with red indicating up-regulation and blue indicating down-regulation. (D) Top 10 KEGG pathway enrichment results of differentially expressed miRNAs, with the MAPK signaling pathway significantly enriched. (E) Volcano map of differentially expressed miRNAs (fold change ≥ 1.5, *P* < 0.05). (F) TCGA database analysis showing significantly lower miR-20a-3p expression in HCC tissues than in normal liver tissues. (G) Kaplan–Meier survival analysis showing a trend of shortened overall survival in HCC patients with low expression of miR-20a-3p precursor (miR-20a). (H) RT-qPCR confirming significantly increased miR-20a-3p expression in IF1-knockdown HCCLM3 cells. (I) RT-qPCR confirming significantly decreased miR-20a-3p expression in IF1-overexpressing HepG2 cells. (J) Immunofluorescence colocalization showing that regions with high IF1 expression (red) had low miR-20a-3p expression (green) (DAPI-stained nuclei, blue). (K) Correlation analysis showing a negative correlation between IF1 and miR-20a-3p expression. Error bars represent mean ± SD. The *t* test was used for statistical analysis (ns, not significant; **P < 0.01, ***P < 0.001).

Then, we extracted RNA from HCCLM3 cells with and without IF1 knockdown and performed transcriptome sequencing (Fig. 3A). The PCA plot showed an appreciable separation between the 2 groups (Fig. 3B). The results were visualized as a heatmap and showed a significant difference of miRNAs expression between the IF knockdown and control groups (Fig. 3C). Meanwhile, the Kyoto Encyclopedia of Genes and Genomes (KEGG) pathway enrichment analysis of miRNA-seq data was performed and revealed several important metabolism and inflammation-related pathways, such as the MAPK signaling pathway (Fig. 3D). According to our results, differential miRNAs were identified through the fold-change values ≥ 1.5 and *P* < 0.05 thresholds, and the most clearly up-regulated miRNA was miR-20a-3p (Fig. 3E). Analysis of TCGA data showed that HCC tissues exhibited the evidently decreased miR-20a-3p levels (Fig. 3F). According to Kaplan–Meier survival analysis, down-regulated miR-20a-3p precursor (miR-20a) expression was related to the unfavorable prognostic outcome of HCC patients (Fig. 3G). Moreover, a previous study reported that down-regulation of miR-20a-3p promoted EMT and tumor growth in pancreatic ductal adenocarcinoma [17]. Next, miRNA reverse transcription polymerase chain reaction (RT-PCR) was performed to detect the changes of miR-20a-3p expression and the results revealed that miR-20a-3p levels were significantly increased after IF1 knockdown in HCCLM3 cells (Fig. 3H) and decreased after IF1 overexpression in HepG2 cells (Fig. 3I). Immunofluorescence staining (Fig. 3J) and correlation analysis (Fig. 3K) results showed that miR-20a-3p expression decreased as IF1 expression increased. Taken together, these findings reveal a negative correlation between IF1 and miR-20a-3p. Integrating the above results, IF1 inhibits the expression of miR-20a-3p in HCC.

### IF1 inhibits miR-20a-3p expression by promoting mitochondrial metabolic reprogramming and mitochondrial ROS accumulation

**Fig. 4.**
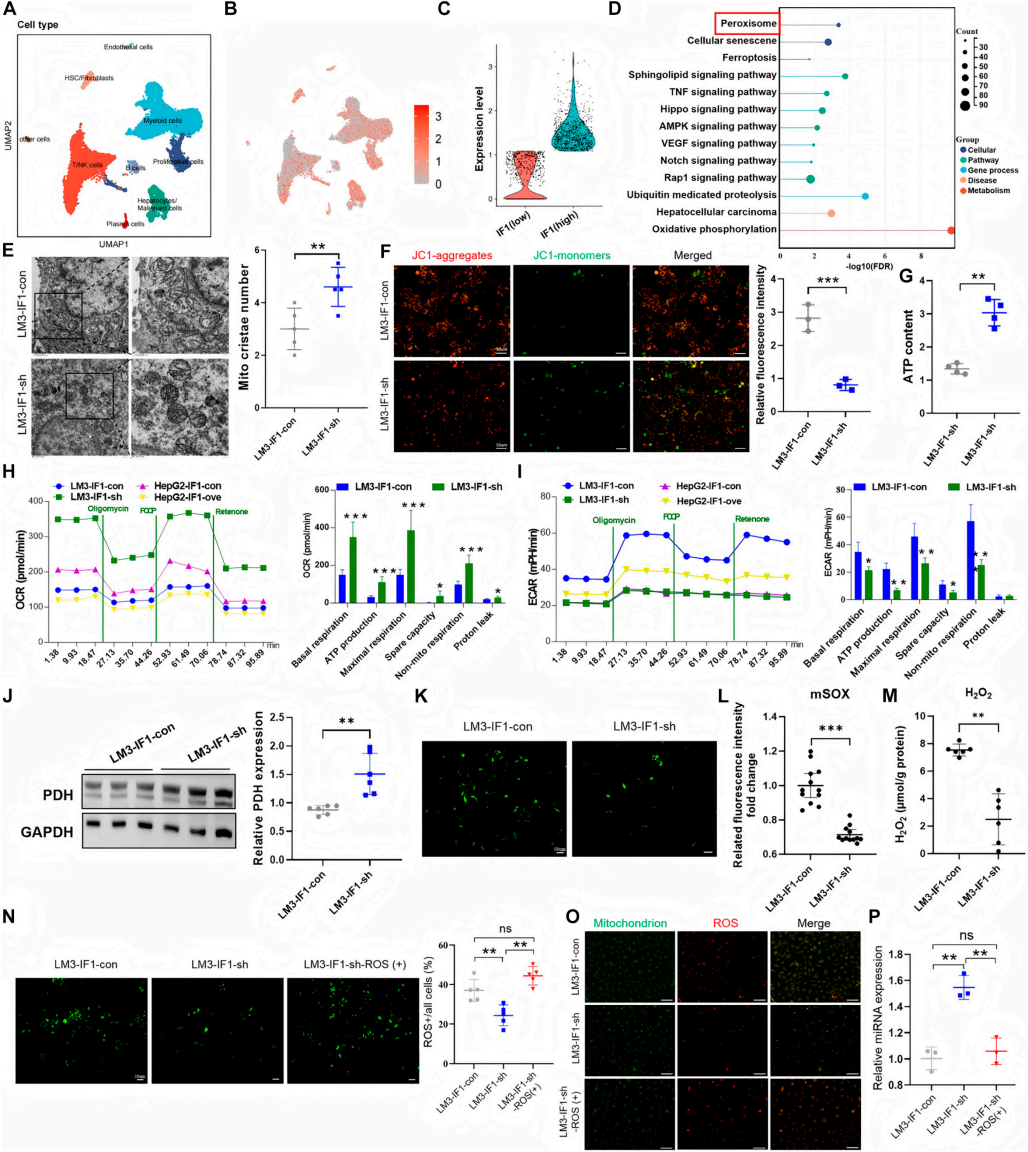
IF1 inhibits miR-20a-3p expression through promoting mitochondrial dysfunction and ROS accumulation. (A) Localization of 9 types of major cells in HCC samples. (B) scRNA-seq analysis showing IF1 expression in all cell types. (C) Malignant cells were divided into high- and low-IF1 expression subgroups based on IF1 levels to screen for DEGs. (D) KEGG enrichment analysis of differentially expressed genes. (E) Transmission electron microscopy showing increased number of intact mitochondrial cristae and improved mitochondrial structure in IF1-knockdown HCCLM3 cells. (F) JC-1 staining showing significantly decreased mitochondrial membrane potential (ratio of JC-1 aggregates to JC-1 monomers) in IF1-knockdown HCCLM3 cells. (G) ATP assay showing significantly increased mitochondrial ATP production in IF1-knockdown HCCLM3 cells. (H and I) Schematic diagrams of OCR (H) and ECAR (I) time courses of cells of different treatment groups cells under basal conditions and following perturbation of mitochondrial respiration with oligomycin, FCCP, and rotenone and statistical analysis. (J) Western blot showing significantly increased expression of PDH, a key enzyme in OXPHOS, in IF1-knockdown HCCLM3 cells. (K) DCFH-DA staining showing significantly decreased total intracellular ROS levels in IF1-knockdown HCCLM3 cells. (L and M) Detection of mitochondrial superoxide (mSOX) and cytoplasmic hydrogen peroxide (H_2_O_2_) showing significant reduction of both in IF1-knockdown HCCLM3 cells. (N and O) Immunofluorescence showing that IF1 knockdown reduced total intracellular ROS (N) and mitochondrial ROS (O) levels in HCCLM3 cells, which could be reversed by adding a mitochondria-specific ROS agonist. (P) RT-qPCR showing that IF1 knockdown significantly increased miR-20a-3p expression, and this effect was reversed by adding a mitochondrial ROS agonist. ROS, reactive oxygen species; OCR, oxygen consumption rate; ECAR, extracellular acidification rate. Error bars represent means ± SD. The *t* test was used for statistical analysis (ns, not significant; *P < 0.05, **P < 0.01, ***P < 0.001).

To investigate the underlying mechanism by which IF1 inhibits the expression of miR-20a-3p, we first analyzed an scRNA-seq dataset reported by Yen et al. [18]. In this dataset, nearly 8,000 cells were subjected to standard quality control, and the qualified cells were selected for the analysis of IF1 function (Fig. S2A). The selected cells were first clustered into 15 subsets (Fig. S2B), the cell population was annotated with previously published marker genes (Fig. 4A and Fig. S2C), and the clustered feature genes for each subpopulation were shown as a heatmap representation (Fig. S2D). The analysis of IF1 expression revealed that IF1 was expressed in all kinds of cells (Fig. 4B). Then, malignant cells were divided into 2 subgroups based on IF1 expression levels (Fig. 4C) to identify the relevant DEGs, which were further subjected to KEGG enrichment analysis. According to the KEGG pathway analysis, we found that peroxisome, which is involved in multiple metabolic processes, including fatty acid oxidation and ROS metabolism [19], was a significantly enriched pathway of interest (Fig. 4D). An increasing number of studies have demonstrated that ROS can regulate miRNA expression mainly by modulating the biogenesis course, transcription factors, and epigenetic changes [20]. Formentini et al. [13] illustrated that IF1 promotes the reprogramming of energy metabolism, including OXPHOS and aerobic glycolysis, which results in ROS generation in colon cancer. Therefore, we hypothesized that IF1 can influence ROS production by changing mitochondrial function and thereby regulate miR-20a-3p expression.

Mitochondrial function has a close relation with organelle structure and morphology. This study first analyzed mitochondrial morphology after IF1 down-regulation and up-regulation. Compared with the control group, IF1-knockdown dramatically promoted cristae array and integrity, while increasing mitochondrial cristae number (Fig. 4E), which indicates improved mitochondrial function by knockdown IF1. In contrast, the morphology of mitochondria was changed significantly when IF overexpression mainly manifested as reduction of mitochondrial ridge (Fig. S3A). We also explored how IF1 affected mitochondrial function of HCC cells and results showed that IF1 knockdown led to decreased mitochondrial membrane potential (Fig. 4F) and increased ATP (Fig. 4G), whereas IF1 overexpression increased mitochondrial membrane potential (Fig. S3B) and reduced ATP (Fig. S3C). Consistent with prior results, IF1 knockdown in HCC cells activated mitochondrial OXPHOS (Fig. 4H) and triggered suppression of glycolysis (Fig. 4I). Furthermore, we detected the expression of pyruvate dehydrogenase complex (PDH), which acted as a key enzyme that promotes OXPHOS, using Western blot and found that the expression of PDH was significantly increased after IF1 knockdown (Fig. 4J). Mitochondrial complexes (Complexes I to V) are the core functional units of OXPHOS. We further investigated the relationship between IF1 and mitochondrial complexes, and Western blot results indicated that after IF1 knockdown, the expressions of mitochondrial Complex I and Complex II were significantly increased (Fig. S3D).

Then, we examined the intracellular ROS content by immunofluorescence; as discovered, the ROS content apparently decreased when IF1 was knocked down (Fig. 4K and Fig. S3E). Meanwhile, we also detected mitochondrial superoxide (mSOX) and cytoplasmic hydrogen peroxide (H_2_O_2_) separately, and found that both were significantly decreased (Fig. 4L and M). We further studied the effect of mitochondrial ROS alterations on miR-20a-3p expression. Immunofluorescence assay revealed that IF1 knockdown reduced the content of intracellular ROS (Fig. 4N) and mitochondrial ROS (Fig. 4O and Fig. S3F). At the same time, the PCR analysis revealed a significant increase of miR-20a-3p expression, whereas treatment with the mitochondria-specific ROS agonist reversed the IF1-mediated changes in miR-20a-3p expression (Fig. 4P). Similarly, IF1 overexpression promoted excessive ROS generation (Fig. S3G and H), including mSOX and cytoplasmic H_2_O_2_ (Fig. S3I). Reduction of mitochondrial ROS reversed the IF1-mediated down-regulation of miR-20a-3p expression (Fig. S3J and K), supporting a role for mitochondrial ROS in IF1-mediated miR-20a-3p expression.

### IF1-induced ROS accumulation inhibits miR-20a-3p expression by promoting DNA methylation of ESR1

**Fig. 5.**
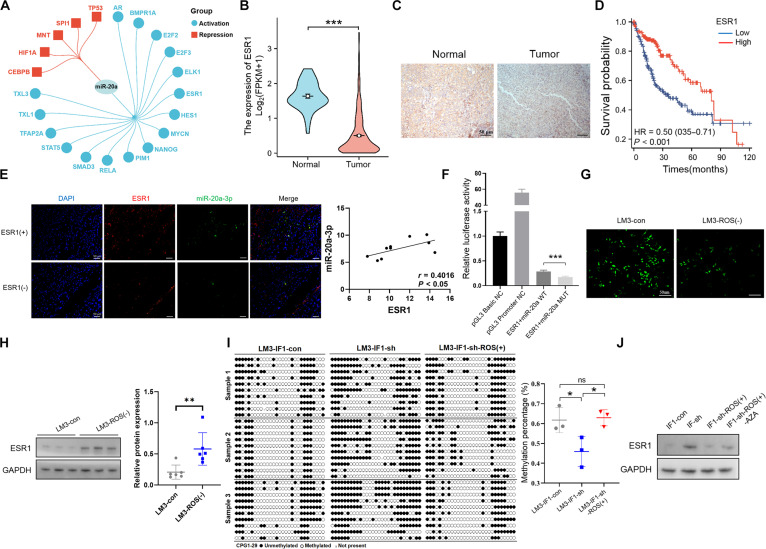
IF1-induced ROS accumulation inhibits miR-20a-3p expression through promoting DNA methylation of ESR1. (A) Prediction of transcription factors for the miR-20a-3p precursor using the TransmiR v2.0 database, identifying 21 candidate factors. (B) TCGA database analysis showing significantly lower ESR1 expression in HCC tissues than in normal liver tissues. (C) IHC staining confirming significantly lower ESR1 protein expression in HCC tissues than in normal liver tissues. (D) Kaplan–Meier survival analysis showing significantly shortened overall survival in HCC patients with low ESR1 expression. (E) Immunofluorescence colocalization showing that regions with high ESR1 expression (red) had high miR-20a-3p expression (green) (DAPI-stained nuclei, blue), and correlation analysis indicating a positive correlation between ESR1 and miR-20a-3p. (F) Dual-luciferase reporter assay showing that ESR1 significantly enhanced the luciferase activity of the wild-type (WT) miR-20a-3p precursor promoter but had no significant effect on the mutant (MUT) promoter, confirming that ESR1 directly regulates miR-20a-3p transcription. (G) Immunofluorescence showing significantly decreased intracellular ROS levels in HCCLM3 cells after treatment with a ROS inhibitor. (H) Western blot showing significantly increased ESR1 protein expression in HCCLM3 cells after treatment with a ROS inhibitor. (I) Methylation-specific PCR showing significantly decreased ESR1 promoter methylation level in IF1-knockdown HCCLM3 cells, which was reversed by adding a ROS agonist. (J) Western blot showing that IF1 knockdown up-regulated ESR1 expression, while adding a ROS agonist decreased ESR1 expression, and treatment with a DNA methylation inhibitor restored ESR1 expression. Error bars represent mean ± SD. The *t* test and ANOVA test were used for statistical analysis (ns, not significant; *P < 0.05, **P < 0.01, ***P < 0.001).

In further experiments, we aimed to explore how IF1-induced mitochondrial ROS accumulation can inhibit miR-20a-3p expression. We used the TransmiR v2.0 database [17] to predict and screen 21 transcription factors of the miR-20a-3p precursor, including 16 activating transcription factors and 5 inhibitory transcription factors (Fig. 5A). Bioinformatics analysis then indicated that, among the activating transcription factors, ESR1 expression levels were clearly lower in HCC tissues than in the surrounding normal tissues (Fig. 5B). We then performed ESR1 IHC staining in HCC tissues and peripheral normal tissues, finding significantly lower ESR1 protein expression levels in HCC (Fig. 5C). Moreover, HCC patients with lower ESR1 expression levels had a significantly worse prognosis than those with higher levels (Fig. 5D), which supported the tumor suppressor function of ESR1. Next, we performed immunofluorescence staining and correlation analysis, which suggested that ESR1 and miR-20a-3p expression levels were positively correlated (Fig. 5E). Dual-luciferase reporter assays suggested that ESR1 can positively regulate miR-20a-3p precursor promoter activity (Fig. 5F). We then further explored whether ROS could regulate ESR1 expression. An ROS inhibitor was used to decrease mitochondrial ROS generation, which further resulted in up-regulated ESR1 expression levels (Fig. 5G). Growing evidence supports a role of ROS-induced oxidative stress in epigenetic processes, including DNA methylation [18]. Therefore, we further investigated whether IF1 could influence the methylation status of the *ESR1* gene. The cytosine-phosphate-guanine (CpG) island in the *ESR1* gene promoter region was predicted using online University of California, Santa Cruz (UCSC) Genome Bioinformatics (Fig. S5A). The *ESR1* gene methylation status was detected in HCC cells by methylation-specific PCR (Fig. S5B). Then, we evaluated the effects of IF1-mediated ROS alteration on the *ESR1* gene promoter methylation level, which revealed that *ESR1* methylation was significantly decreased after IF1 knockdown, which was reversed with increased ROS (Fig. 5I). Generally, DNA methylation in the promoter region of a gene represses its transcription. We further evaluated the effects of IF1-mediated ROS alteration on ESR1 expression by Western blot analysis. IF1 knockdown inhibited ESR1 protein expression, which was reversed with the increase in ROS levels. However, re-expression of ESR1 was induced following treatment with a DNA methylation inhibitor (Fig. 5J). These results suggest that IF1-induced ROS accumulation can inhibit the *ESR1* gene methylation level, thereby promoting the expression of ESR1.

### IF1 promotes HCC VM formation, tumor development, and lung metastasis by inhibiting miR-20a-3p

**Fig. 6.**
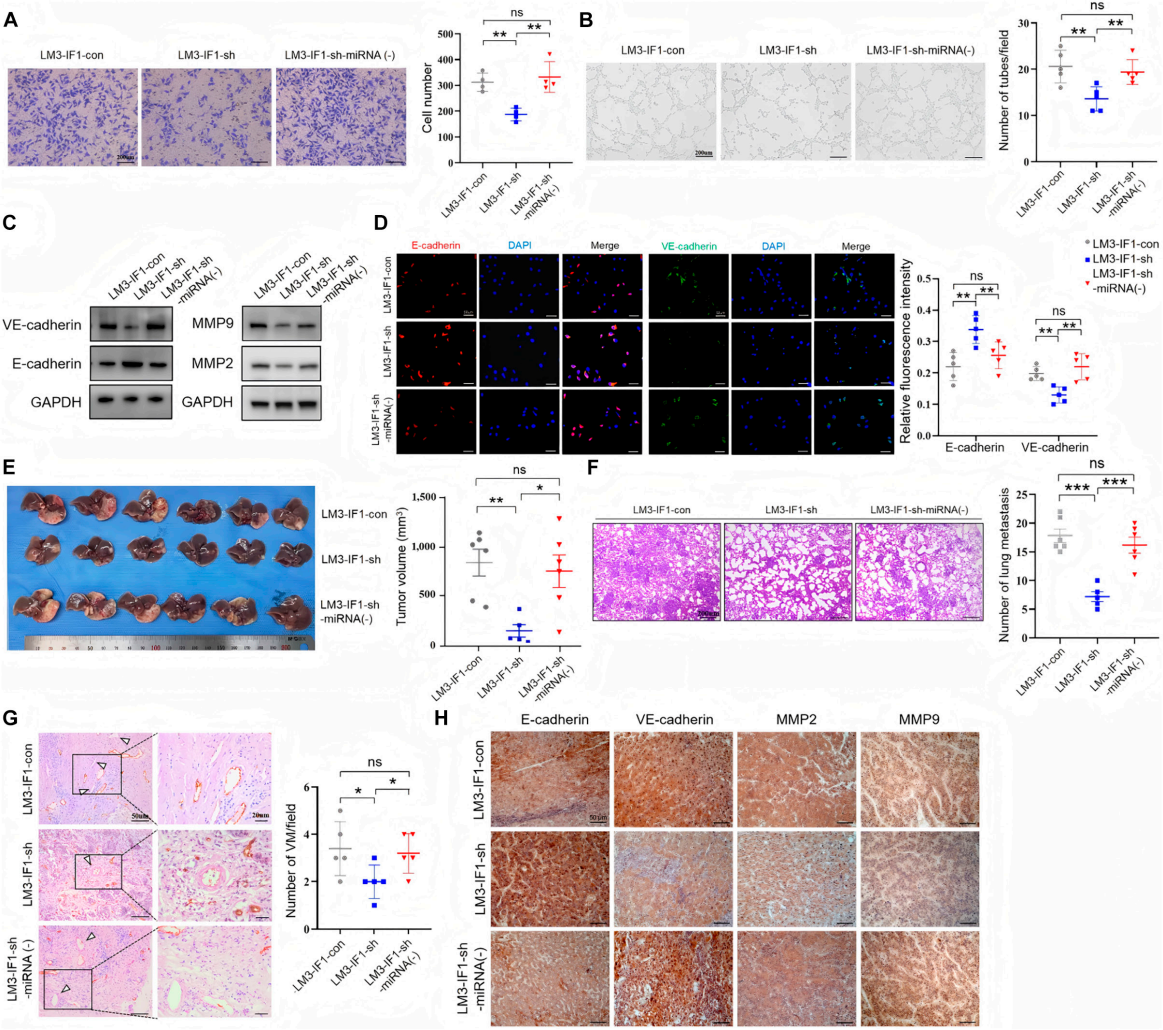
IF1 promotes HCC VM formation, tumor growth, and lung metastasis by inhibiting miR-20a-3p. (A) Transwell migration assay showing that IF1 knockdown significantly reduced the migration ability of HCCLM3 cells, and cotransfection with a miR-20a-3p inhibitor reversed this effect. (B) Tube formation assay showing that IF1 knockdown significantly decreased the number of tube-like structures formed by HCCLM3 cells, and cotransfection with a miR-20a-3p inhibitor restored tube formation ability. (C) Western blot showing that IF1 knockdown up-regulated E-cadherin and down-regulated VE-cadherin/MMP2/MMP9 in HCCLM3 cells, and cotransfection with a miR-20a-3p inhibitor reversed this protein expression trend. (D) Immunofluorescence showing that IF1 knockdown increased E-cadherin and decreased VE-cadherin in HCCLM3 cells, and cotransfection with a miR-20a-3p inhibitor restored their expression. (E and F) Orthotopic HCC model experimental analyses including images of tumors in each group, tumor volume (E), and the number of lung metastases (F) to demonstrate HCC cell growth and metastasis in vivo (*n* = 6 each group). (G) CD31/PAS staining showing significantly lower VM density in tumors of the IF1-knockdown group, and cotransfection with a miR-20a-3p inhibitor restored VM density. (H) IHC staining showing that the IF1-knockdown group had up-regulated E-cadherin and down-regulated VE-cadherin/MMP2/MMP9 in tumors, and cotransfection with a miR-20a-3p inhibitor reversed this expression pattern. Error bars represent means ± SD. The ANOVA test was used for statistical analysis (ns, not significant; *P < 0.05, **P < 0.01, ***P < 0.001).

We further examined whether IF1 can modulate HCC VM by regulating miR-20a-3p. We overexpressed miR-20a-3p in IF1-overexpression HepG2 cells (Fig. S5A) and knocked down miR-20a-3p with lentivirus in IF1-knockdown HCCLM3 cells (Fig. S5B). As expected, IF1 knockdown significantly suppressed the invasion and tube formation of HCCLM3 cells, while down-regulation of miR-20a-3p reversed this effect (Fig. 6A and B). Additionally, Western blot analysis showed that IF1 knockdown up-regulated the protein expression levels of E-cadherin and down-regulated those of VE-cadherin, MMP2, and MMP9 with miR-20a-3p knockdown reversing the effects of IF1 knockdown in HCCLM3 cells (Fig. 6C and Fig. S5C). Immunofluorescence data further confirmed these findings (Fig. 6D). Next, to investigate if miR-20a-3p could reverse IF1-mediated tumorigenesis and VM formation in vivo*,* we established an orthotopic liver tumor model. Mice injected with IF1-knockdown HCCLM3 cells had lower tumor weights (Fig. 6E) and fewer lung metastases (Fig. 6F). These trends were reversed by miR-20a-3p down-regulation. Moreover, miR-20a-3p knockdown attenuated the IF1 knockdown-mediated decrease in HCC VM density (Fig. 6G). Within tumors, miR-20a-3p also promoted VE-cadherin, MMP-2, and MMP-9 expression, but inhibited E-cadherin expression (Fig. 6H). Similarly, IF1 overexpression significantly increased the HepG2 cell invasion ability and tube formation, while up-regulation of miR-20a-3p reversed these effects (Fig. S5D and E). IF1 overexpression promoted the expression of VE-cadherin, MMP-2, and MMP-9 and suppressed that of E-cadherin, with miR-20a-3p overexpression reversing these effects in HepG2 cells (Fig. S5F). Immunofluorescence data further confirmed these findings (Fig. S5G). Mice injected with IF1-overexpression HepG2 cells had higher tumor weight (Fig. S6A) and more lung metastases (Fig. S6B), which were reversed by injection of miR-20a-3p overexpression vectors. Moreover, up-regulation of miR-20a-3p attenuated the effects of IF1 overexpression on increased VM density in HepG2 tumors (Fig. S6C). Furthermore, IHC staining of tumor tissues corroborated the Western blot analysis data (Fig. S6D). In summary, miR-20a-3p is involved in IF1-mediated HCC progression and VM formation.

### GNAZ, as a target of miR-20a-3p, promotes HCC cell tube formation

**Fig. 7.**
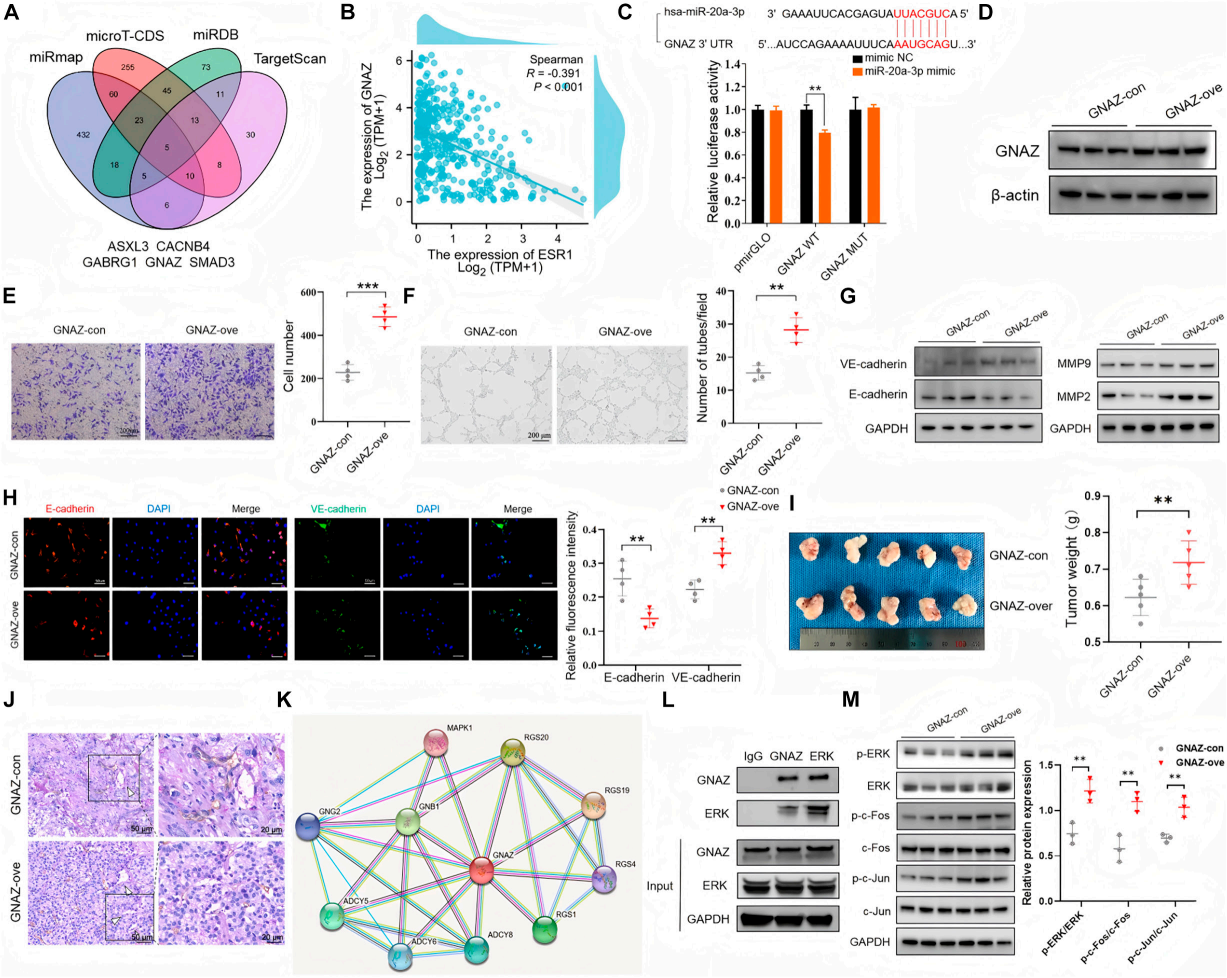
GNAZ is a direct target of miR-20a-3p and can promote tumor growth and VM of HCC. (A) Prediction of miR-20a-3p target genes using 4 databases (microT-CDS, miRDB, TargetScan, and miRmap), identifying 5 candidate genes through intersection. (B) Correlation analysis from the TCGA database showing a negative correlation between ESR1 and GNAZ expression. (C) Dual-luciferase reporter assay showing that miR-20a-3p mimics significantly inhibited the luciferase activity of the GNAZ WT reporter gene but had no effect on the MUT reporter gene, confirming that GNAZ is a direct target of miR-20a-3p. (D) Western blot confirming significantly increased GNAZ protein expression in HepG2 cells transfected with a GNAZ overexpression plasmid. (E) Transwell migration assay showing significantly enhanced migration ability of HepG2 cells after GNAZ overexpression. (F) Tube formation assay showing significantly increased number of tube-like structures formed by HepG2 cells after GNAZ overexpression. (G) Western blot showing that GNAZ overexpression down-regulated E-cadherin and up-regulated VE-cadherin/MMP2/MMP9 in HepG2 cells. (H) Immunofluorescence showing that GNAZ overexpression decreased E-cadherin and increased VE-cadherin in HepG2 cells. (I) Orthotopic HCC model experimental analyses including images of tumors in each group, and tumor weight to demonstrate HCC cell growth in vivo (*n* = 5 for each group). (J) CD31/PAS staining showed VM density in HCC tissues (*n* = 5 for each group). (K) Schematic representation of the protein–protein interaction network centered on GNAZ and its associated proteins. (L) Coimmunoprecipitation assay showing the interaction between GNAZ and ERK. (M) Western blot analysis of the protein levels of p-ERK, ERK, p-c-Fos, c-Fos, p-c-Jun, and c-Jun in cells with GNAZ overexpression compared to control. The protein level was calculated by ImageJ. Error bars represent means ± SD. The *t* test and ANOVA test were used for statistical analysis (ns, not significant; **P < 0.01, ***P < 0.001).

To better understand the potential molecular mechanisms through which miR-20a-3p regulates VM in HCC, we next explored the target protein of miR-20a-3p. Target proteins of miR-20a-3p were predicted using 4 online databases: microT-CDS (http://www.microrna.gr/microT-CDS), miRDB (https://mirdb.org/), TargetScan (https://www.targetscan.org/vert_80/), and miRmap (https://mirmap.ezlab.org/). By intersecting prediction results from these databases, 5 candidate mRNAs were selected: Additional sex combs like 3 (ASXL3), Calcium voltage-gated channel auxiliary subunit beta 4 (CACNB4), Gamma-aminobutyric acid type A receptor subunit gamma 1 (GABRG1), GNAZ, and SMAD family member 3 (SMAD3) (Fig. 7A). Next, we analyzed the gene expression level of the 5 target molecules and their correlations with the prognosis of HCC patients. TCGA data showed that GNAZ expression clearly increased in tumor relative to surrounding non-carcinoma sample (Fig. S7A). Moreover, HCC patients with higher GNAZ expression had a significantly worse prognosis than those with lower levels (Fig. S7B), which suggest that GNAZ acts as a tumor-promoting gene in HCC. Correlation analysis revealed a significant strong correlation between GNAZ and VM and VM micro-environment-associated markers (Fig. S7C). Significantly, the expression level of ESR1 was negatively correlated with GNAZ expression (Fig. 7B). Therefore, we examined whether GNAZ was a direct target gene of miR-20a-3p using a luciferase reporter assay, and results indicated that overexpression of miR-20a-3p significantly inhibited luciferase activity in GNAZ-WT, but not GNAZ-MUT (Fig. 7C). We performed Western blot analysis and found that IF1 overexpression enhanced GNAZ expression in HepG2 cells (Fig. S7D), while IF1 knockdown in HCCLM3 cells decreased GNAZ levels (Fig. S7E). In addition, validation experiments revealed that miR-20a-3p overexpression significantly decreased GNAZ expression levels in HepG2 cells (Fig. S7F). In summary, GNAZ is the direct target of miR-20a-3p in HCC. To investigate whether GNAZ dysregulation can affect HCC VM formation, GNAZ was transiently overexpressed in HepG2 cells by transfection of an expression plasmid (Fig. 7D and Fig. S7G). GNAZ overexpression significantly increased HepG2 cell invasion ability (Fig. 7E) and tube formation (Fig. 7F). Furthermore, we investigated the expression of VM formation-associated markers and found that GNAZ overexpression suppressed E-cadherin expression and increased that of VE-cadherin, MMP2, and MMP9 (Fig. 7G and Fig. S7H), as evidenced by immunofluorescence analysis (Fig. 7H). Moreover, the mice injected with the GNAZ-overexpressed HepG2 cells showed increasing tumor weight (Fig. 7I) and VM density (Fig. 7J) than the controls. Collectively, GNAZ makes a great contribution to HCC VM.

Next, to uncover the mechanism involved in GNAZ-mediated enhancement of invasion and VM formation of HCC, we applied a bioinformatics tool to screen for novel GNAZ-interacting proteins. The STRING database, a web interface for gene function analysis, predicted interactions between GNAZ and partner proteins (Fig. 7K). Among the predicted partner proteins, MAPK1 (ERK2) serves as a key component in MAP kinase pathway, which has a critical effect on cell growth, apoptosis, angiogenesis, differentiation, and tumor metastasis [21]. Thus, we hypothesized that GNAZ directly controls activation of the ERK signaling pathway. First, we identified an interaction between GNAZ and ERK using coimmunoprecipitation combined with Western blot (Fig. 7L). Then, Western blot analysis demonstrated that GNAZ overexpression led to increased ERK phosphorylation, as well as increased phosphorylation of downstream transcription factors, including c-Jun and c-Fos (Fig. 7M), which are important in VM formation. Finally, tube formation assays showed that inhibition of ERK expression could reverse the increased tube formation induced by GNAZ overexpression (Fig. S7I). Together, these observations suggest that GNAZ promotes HCC VM by phosphorylating the ERK pathway.

## Discussion

**Fig. 8.**
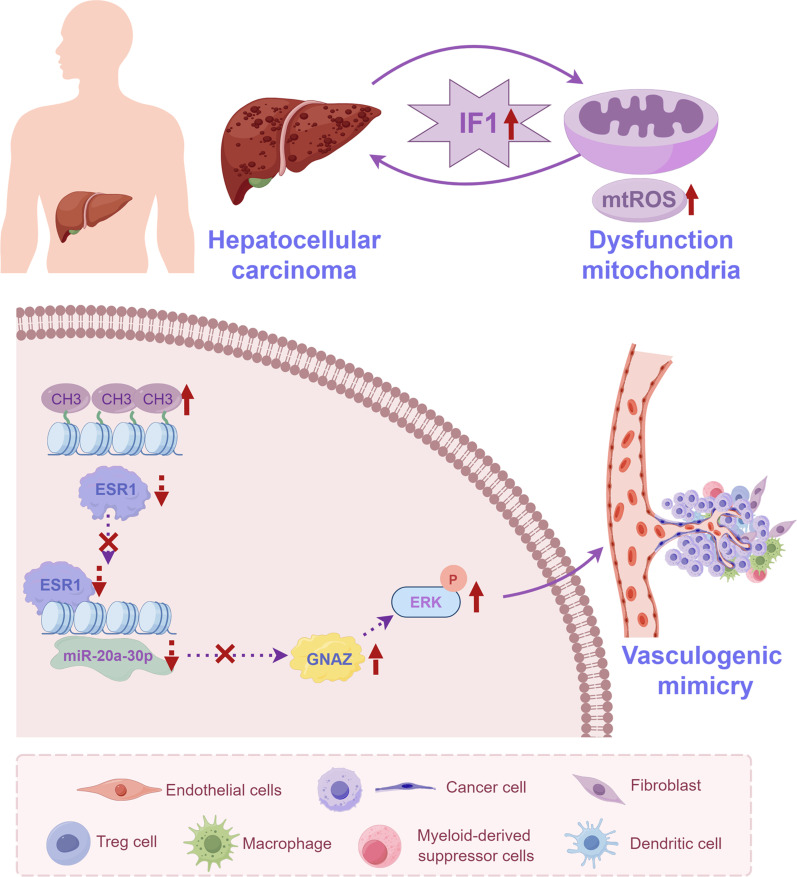
A sketch map showing the molecular mechanism of IF1 in promoting HCC VM. ATPase inhibitory factor 1 drives mitochondrial energy metabolic reprogramming to promote HCC vasculogenic mimicry via the ESR1/miR-20a-3p/GNAZ pathway.

Given the relationship between tumor aggressiveness and VM, seeking therapies to target VM using genesis and progression mechanisms is of great importance [20]. In this study, we found that markedly increased IF1 expression patterns in HCC contribute to tumor growth and VM formation via the ESR1 and miR-20a-3p/GNAZ/ERK pathway (Fig. 8). These findings may point to new strategies to prevent HCC progression and metastasis.

Therapies targeting angiogenesis have been extensively applied in HCC, with encouraging clinical outcomes reported. However, these treatments have only moderately improved overall survival rates, with no persistent responses [22]. VM represents a newly recognized mechanism of angiogenesis found in various cancerous tumors, distinguishing it from traditional angiogenic processes driven by the vascular endothelium. VM involves structures resembling blood vessels that are created by tumor cells and the ECM, which supplies nutrients to tumors [6]. Currently, there is ample evidence supporting close relationships between VM and high tumor aggressiveness, tumor recurrence, and hematogenous metastasis rates, as well as with decreased survival rates and unfavorable prognostic outcomes of HCC patients [7,21,23]. Consistent with previous findings, we also observed VM occurrence within HCC tissues in this study.

Research has increasingly demonstrated that metabolic reprogramming is related to disease progression, clinical outcomes, and treatment responses in a range of cancers [24]. Proliferating cells exhibit a metabolic shift from OXPHOS to glycolysis [25]. According to recent findings, numerous human tumor types display elevated IF1 expression levels, which can suppress mitochondrial ATP synthase activity and are important for the metabolic change in cancer cells toward increased aerobic glycolysis [13,26]. IF1 promotes endothelial-dependent angiogenesis and acts as an independent prognostic factor in HCC [15]. However, no previous study has illustrated the connection between IF1 and VM. Here, samples from 35 HCC patients were analyzed by IHC staining, which exhibited IF1 overexpression and a positive correlation with VM, suggesting that IF1 may be important in VM.

Numerous studies have also demonstrated that the EMT can endow tumor cells with stem cell-like characteristics. Notably, tumor cells capable of VM can mimic endothelial cells as a type of mesenchymal cell, similar to the EMT process [27–31]. VM is closely related to the EMT process in HCC, with various known EMT inducers participating in VM development and regulation in this context [28]. In this study, the effects of IF1 on tumor invasion and metastasis were directly demonstrated in both our in vitro and in vivo experiments, with correlations observed between IF1 expression and markers of the EMT and VM in HCC tissues and cells. IF1 expression levels were positively related to those of VE-cadherin, vimentin, MMP2, and MMP9, while they were negatively related to E-cadherin, indicating that IF1 can promote tumor progression by enhancing the EMT and VM formation.

Research has demonstrated that miRNAs are related to disease development and progression [29–31]. There is increasing evidence suggesting that miRNA dysregulation contributes to increased cell migration, invasion, and angiogenesis and reduced dedifferentiation and apoptosis that ultimately support tumorigenesis [32]. The EMT exerts significant effects on VM in HCC, with certain miRNAs being implicated in the EMT process that facilitate regulation of VM in HCC. For example, Zhao et al. performed a miRNA microarray using HCC cells and demonstrated that Bcl-2 boosts Twist-1 functions in VM and the EMT via miRNAs [33]. In HCC, VM involves various signaling pathways, like IL-6–STAT3, PI3K/AKT, and Notch4 [6]. Hence, miRNAs that block these pathways can suppress VM during HCC. Here, we performed miRNA sequencing and found that IF1 was closely associated with miR-20a-3p expression patterns. Ma et al. confirmed that circ-0005105 expression levels are markedly elevated in pancreatic ductal adenocarcinoma tissues and are related to unfavorable prognostic outcomes. Mechanistically, circ-0005105 promotes COL11A1 expression by sponging miR-20a-3p to activate the EMT [17,34–37]. Results of our current in vitro and in vivo work imply that miR-20a-3p is responsible for IF1-mediated EMT and VM. Prediction of miRNA target genes is important, as miRNAs execute their regulatory functions primarily through modulating target mRNA levels. Finally, we identified GNAZ as the downstream target of miR-20a-3p using biological information databases and dual-luciferase assays. G proteins play important roles in cellular signaling by connecting activated 7-helix receptors to effector molecules. GNAZ is also known as the Gαz subunit of the Gz protein, with evidence suggesting that Gz is involved in regulating particular MAPK subfamilies [38]. Consistently, our results revealed that GNAZ can promote ERK phosphorylation. Furthermore, according to Tian and Cai [39], GNAZ up-regulation was linked to unfavorable outcomes and enhanced cell cycle progression at the G0/G1 phase of HCC. In this study, our results provide new data supporting a role for GNAZ in HCC EMT and VM.

Many tumor-promoting processes, such as oncogene activation, tumor suppressor loss-of-function, mitochondrial function alterations, changed stromal interactions, and promoted hypoxia, can enhance ROS generation [40]. ROS affect tumor cell and stromal component behaviors for modulating tumor cell survival and progression [41]. IF1 overexpression inhibits mitochondrial ATP synthase activity and triggers mitochondrial hyperpolarization and subsequent ROS production, which can enhance colon cancer cell proliferation and survival through NF-κB pathway activation [13]. Moreover, the disparate cancer-related signal-induced increase in mitochondrial ROS and membrane ROS can induce the EMT [42,43]. Similarly, we also observed a role for IF1 in ROS production in HCC. In addition, we found that ROS are critically involved in IF1-mediated miR-20a-3p expression.

Overall, IF1-induced mitochondrial energy metabolic reprogramming drives DNA methylation of ESR1 to promote HCC VM via the miR-20a-3p/GNAZ/ERK pathway. Overall, our data suggest that targeting IF1 and miR-20a-3p to disrupt VM is a potential therapeutic approach for treating HCC. In subsequent studies, we will collaborate with the medicinal chemistry team to develop IF1 competitive inhibitors, verify their efficacy in inhibiting VM while ensuring safety for normal liver tissue, and provide tools for targeted therapy

## Materials and Methods

### Identification of VM-related DEGs

RNA sequencing data of HCC samples and normal liver tissues were obtained from the TCGA database (https://portal.gdc.cancer.gov). The SangerBox tool (http://sangerbox.com/) was used to normalize gene expression data before further analysis. The related genes of VM, aerobic glycolysis, hypoxic TME, EMT, and ECM remodeling were obtained from the GeneCards Database (https://www.genecards.org)

### Patient samples and ethics

This study recruited a total of 35 patients with HCC without other severe organ diseases who had not received antiplatelet drug application, chemotherapy, or interventional therapy from May 2020 to May 2022 at the Affiliated Beijing Chaoyang Hospital of Capital Medical University (Table S6). HCC diagnosis was based on World Health Organization criteria [44]. Written informed consent was obtained from each patient included in the study. All human tissues were collected according to Health Insurance Portability and Accountability Act (HIPAA) compliant protocols, and the samples were used in accordance with the standards of the center’s ethics committee (Ethics number: 2024-K-315).

### Cell culture

Human HCC cell lines (HepG2, HCCLM3, SMMC7721, and Huh-7) were provided by Cell Resource Center, Chinese Academy of Medical Sciences, Peking Union Medical College. The immortalized LO2 liver cells were supplied by the Institute of Biochemistry and Cell Biology at the Chinese Academy of Sciences in China. They were cultured in Dulbecco’s Modified Eagle Medium (DMEM; HyClone, Logan, UT, USA) with 10% fetal bovine serum (Gibco, Waltham, MA, USA). All cells were maintained in an incubator at 37 °C with a 5% CO_2_ atmosphere.

### Transfection experiments

Lentiviral-mediated short hairpin RNA IF1 (CACCATGAAGAAGAAATCGTT) and lentiviral vectors encoding the human IF1 gene (NM_016311.4) were constructed by LiKeli BioTECH Co. Ltd. (Beijing, China). After reaching 40% to 50% confluency, the cells were inoculated into medium prior to transfection using polybrene A reagent and lentivirus following specific protocols. Empty vector was used as a negative control. Twelve hours post-transfection, freshly prepared medium was added. Three days later, a fluorescence microscope was used to observe green fluorescent protein levels, with 2 μg/mL puromycin added for cell selection. The IF1 protein expression levels were analyzed by Western blot analysis.

miR-20a-3p lentivirus and miR-20a-3p inhibitor lentivirus were constructed by Syngentech Co., Ltd. (Beijing, China). Polybrene (Syngentech) was used for infection, with a >80% lentivirus infection efficiency being deemed successful. At 48 h post-infection, the cells were digested to conduct subsequent culture. Each experiment was performed following specific protocols. miR-20a-3p expression levels were analyzed using qRT-PCR.

The GNAZ overexpression plasmid was chemically synthesized in the Laboratory of RNA Chemistry (Syngentech, Beijing, China) before transfection into HepG2 cells using Lipofectamine 3000 reagent (Invitrogen, Carlsbad, CA, USA) following specific protocols. GNAZ protein expression levels were analyzed by Western blot analysis.

### Animal model

We purchased the 4- to 6-week-old male BALB/c nu/nu mice in Vital River Laboratories (Beijing, China) and raised them in the defined flora environment within sterile microisolator cages with individual ventilation. These animal experiments gained approval from the Institutional Animal Care and Use Committee of Capital Medical University (Ethics number: 2024-D-186). We also generated both orthotopic and subcutaneous HCC models. In the subcutaneous HCC tumor model, HCC cells (5 × 10^6^) in 250 μl of serum-free DMEM were injected subcutaneously into the upper right flanks of nude mice. In the orthotopic HCC model, mice were given injection of a 30-μl suspension containing HCC cells in the subcapsular region of the median liver lobe parenchyma, according to previous depiction [45]. When the animal experiment reached the preset endpoint (4 weeks after the establishment of the nude mouse orthotopic HCC model, or when obvious tumor-bearing symptoms appeared, such as a sudden weight loss of >20%, a significant decrease in activity ability, or a tumor diameter exceeding 1.5 cm), carbon dioxide (CO₂) inhalation was used for euthanasia: Nude mice were placed in a closed euthanasia chamber, and CO₂ (purity ≥99.9%) was slowly injected at a rate of 50% volume per minute. After maintaining ventilation for 5 min, the death of the animals was confirmed by palpating the heartbeat, observing the cessation of breathing, and the disappearance of the corneal reflex—ensuring the euthanasia process was painless, rapid, and in line with the standards recommended by the International Council for Laboratory Animal Science. Immediately after euthanasia, tumor tissues, lung tissues, and other samples were dissected and collected for analysis. Tumor volume was calculated using the following formula: tumor volume = (length × width^2^)/2.

### IHC and CD31/PAS double staining

Tissue samples were subjected to formalin fixation and paraffin embedding, and later sectioning. After sequential deparaffinization with xylene, sections were subjected to gradient ethanol hydration, and antigen retrieval was conducted using citrate buffer (pH 6) at high temperature. Samples were then blocked with 10% normal goat serum (ZSGB-BIO, Beijing, China) for 30 min and incubated with primary antibody overnight at 4 °C. After washing with PBS, slides were incubated with secondary antibody (ZSGB-BIO, Beijing, China) for 1 h, and 3,3ʹ-diaminobenzidine (DAB) peroxidase substrate was added for staining (ZSGB-BIO, Beijing, China) until the desired intensity was achieved. For CD31/PAS double staining, sections were stained with CD31, washed with PBS for 3 to 5 min, and then stained using a PAS Kit (Solarbio, Beijing, China). Finally, all sections were counterstained with hematoxylin, dehydrated, and mounted. According to the proportion of positively stained tumor cells from 5 high-power fields (×200), the sections were scored as follows: (0) no positive tumor cells; (1) <25% positive tumor cells; (2) <50% positive tumor cells; (3) <75% positive tumor cells; and (4) > 75% positive tumor cells.

### Western blotting assay

After lysis using RIPA lysis buffer (Solarbio, Beijing, China) that contained protease/phosphatase inhibitors, protein contents within cell lysates were analyzed by the bicinchoninic acid protein assay kit (KeyGEN, Nanjing, China). Later, protein aliquots (20 to 30 μg) underwent separation through 8% to 15% SDS-PAGE prior to transfer onto polyvinylidene fluoride membranes. After 1 h of soaking within 5% nonfat milk, membranes received overnight primary antibody (Table S7) incubation under 4 °C. Later, membranes received 1 h of probing using horseradish peroxidase-conjugated secondary antibodies under ambient temperature. SuperSignal West Pico substrate (Thermo Scientific, Waltham, MA, USA) was employed for detecting immunoreactivity. The bands in different wells are from different groups of samples processed in the same batch; GAPDH and β-actin serve as the internal control, ensuring the traceability of the experimental operation.

### Real-time PCR

After extracting total RNA, it was analyzed by reverse-transcribed quantitative PCR (RT-qPCR) as previously reported [46]. RNA and miRNA were synthesized in cDNA with SuperScript III First-Strand Synthesis Kit (Invitrogen, Carlsbad, CA, USA) and miRcute Plus miRNA First-Strand cDNA Kit (Tiangen, Beijing, China), separately, following specific protocols. Later, the Bio-Rad C1000 Thermal Cycler was utilized to amplify cDNA with SYBR green PCR master mix (Applied Biosystems, USA) and miRNA SYBR green PCR master mix (Tiangen, Beijing, China) for 40 to 45 cycles following specific protocols. The 2^−ΔΔCT^ approach was utilized to determine RNA expression, which was defined by fold-change values relative to controls. Primers for IF1, miR-20a-3p, GNAZ, GAPDH, and U6 were synthesized by Tsingke Biotech (Beijing, China); the sequences were as follows: IF1, 5′-GCGATTATGCCCCTTTCATT-3′ (forward, F) and 5′-CAGCCTGCAGGAAGCT-3′ (reverse, R); miR-20-3p, 5′-ACUGCAUUAUGAGCACUUAAAG-3′ (F); GNAZ, 5′-TAAATAAAAACAAAGCAGAAAACCC-3′ (F) and 5′-CTCTGCTCCAAAGAAATTTTGG-3′ (R); GAPDH, 5′-GCAAAGTGGAGATTGTTGCCAT-3′ (F) and 5′-CCTTGACTGTGCCGTTGAATTT-3′ (R); and U6, 5′-GCTTCGGCAGCACACATACTAAAAT-3′ (F).

### Transwell assay

We conducted Transwell assays with the modified Boyden chamber (Costar-Corning, New York, USA), in which the 24-well plates were coated by polycarbonate filter inserts (pore size, 8.0 μm), according to previous depiction [46].

### Immunofluorescence

After seeding HCC cells onto coverslips and culturing for a 24-h duration, cells were rinsed by PBS, followed by 15 min of immersion within 4% paraformaldehyde and additional 1 h of blocking using 5% bovine serum albumin. Next, the cells underwent overnight incubation using anti-E-cadherin and anti-vimentin antibodies under 4 °C, prior to further 1 h of probing using fluorescence-labeled secondary antibodies under ambient temperature. 4ʹ,6-diamidino-2-phenylindole (DAPI) was used to visualize nuclei. The Leica DMRA fluorescence microscope (Rueil-Malmaison) was employed for image capturing.

### Three-dimensional culture

Matrigel, reduced for growth factor concentration (10 mg/ml; BD Biosciences), was slowly thawed at 4 °C overnight. Subsequently, 70 μl was dispensed into each well of a 96-well plate and permitted to solidify for 30 min at 37 °C. HCC cells (1 × 10^4^) were added to plates and incubated for 4 h. Then, 4 random fields were observed, and the number of tubes was counted. Experiments were repeated independently 3 times. Tube formation was observed, and the total length and numbers of junctions of the tubes were quantified using ImageJ software.

### Vector construction, stable transfection, and dual-luciferase reporter gene assay

Potential miR-20a-3p and GNAZ binding sites were predicted using the starBase 3.0 online tool (http://starbase.sysu.edu.cn/index.php). The whole GNAZ sequence and mutated GNAZ start codon were cloned into the pmirGLO (Tsingke, Beijing, China) vector. These constructs (0.15 μg) were cotransfected into target cells in 96-well plates together with 20 μM miR-20a-3p inhibitor or mimic using Hieff Trans Liposomal Transfection Reagent (YEASEN, Shanghai, China). Cells that had been transfected were collected for the specified analyses 24 h after the transfection. The dual-luciferase reporter assay utilized 293T cells, a luminescent reaction solution (Galen, Beijing, China), and a multifunctional microplate reader (Tecan, Beijing, China). Measurements of the firefly luciferase readings were taken, and the corrected relative luciferase activity was calculated by dividing the appropriate fluorescence value based on the characteristics of the vector.

### Small RNA-seq

Small RNA-seq was conducted in line with the manufacturer’s technical procedures. For specific operation steps, please refer to the previously published literature [47]. Briefly, total RNA was isolated from samples using appropriate extraction kits. The purified RNA underwent adaptor ligation at both the 3′ and 5′ ends, followed by reverse transcription to generate cDNA. PCR amplification was then performed to enrich the cDNA library, which was quality checked using a bioanalyzer. The prepared library was sequenced on a high-throughput sequencing platform (Illumina). Raw sequencing reads were processed by removing adaptors and low-quality sequences. Clean reads were aligned to the reference genome or miRNA database for identification of known miRNAs and prediction of novel ones.

### Mitochondrial respiration assay

To monitor mitochondrial OXPHOS activity, transfected HCC cells were seeded onto a Seahorse XF24 Cell Culture Microplate (Agilent Technologies, Santa Clara, CA, USA) at a density of 5×10^4^ cells per well and allowed to attach in complete medium at 37 °C and 5% CO_2_ for 12 h. Subsequently, the cells were washed twice and incubated in XF base medium (Agilent Technologies) containing 1 mM sodium pyruvate, 4 mM L-glutamine, and 4.5 g/L glucose. A Seahorse XF24 Analyzer (Agilent Technologies) was used to measure the mitochondrial oxygen consumption rate (OCR). The following were injected at the listed final concentrations: oligomycin (1 μM), carbonyl cyanide 4-(trifluoromethoxy) phenylhydrazone (FCCP, 1 μM), antimycin A (0.5 μM), and rotenone (0.5 μM). The basal OCR was measured after the addition of rotenone A was subtracted from the value prior to adding oligomycin. The maximal OCR was measured after the addition of rotenone was subtracted from the value in the presence of FCCP.

### ROS measurement

ROS production was assessed by the conversion of 2′,7′-dichlorofluorescein diacetate (H2DCF-diacetate) into the fluorescent compound 2′,7′-dichlorofluorescein (DCF). Cells were seeded in 6-well plates at 1 × 10^6^ cells per well, allowed to attach overnight, treated with DCF-DA (20 μM) for 40 min at 37 °C in the dark, and washed twice with PBS. Then, the intracellular DCF fluorescence intensity was observed using an Evos microscopy imaging system. Cell brightness was scored visually and compared among the groups.

### Specific regulation and detection of mitochondrial ROS

Mitochondrial ROS inhibitor Mito-TEMPO (SML0737, Sigma-Aldrich) was dissolved in dimethyl sulfoxide (DMSO) to prepare a 10 mM stock solution, which was stored at −20 °C in the dark. Mitochondria-targeted hydrogen peroxide inducer Mito-peroxide (ab141445, Abcam) was dissolved in DMSO to prepare a 5 mM stock solution, which was stored at −20 °C in the dark. mSOX levels were measured using the MitoSO Red Mitochondrial Superoxide Detection Kit (Beyotime, S0061S) following the manufacturer’s instructions. Cells were seeded into 96-well plates, and all groups were incubated with 5 μM MitoSOX working solution for 30 min at 37 °C in the dark. After 3 washes with prewarmed PBS, fluorescence intensity was detected using a microplate reader at the excitation/emission wavelength of 405/610 nm.

### Cell H_2_O_2_ assay

Cells were lysed with lysis buffer and subsequently centrifuged at 12,000×*g* for 5 min at 4 °C. The resulting supernatant was collected, and cell H_2_O_2_ levels were determined following the manufacturer’s instructions provided with the commercial kit (Beyotime, S0038).

### DNA methylation analysis

DNA methylation analysis of ESR1 was performed as previously described [48]. In brief, genomic DNA was obtained from HCC cells using the Trelief Animal Genomic DNA Kit (TsingKe, Beijing, China), then bisulfite-modified using the EpiTect Fast DNA Bisulfite Kit (Qiagen, Valencia, CA, USA). The gene CpG island was predicted online using UCSC Genome Bioinformatics (http://www.genome.ucsc.edu/). The primers used in bisulfite-specific PCR (BSP) detection were designed as follows: F: 5′-GTGTTTGGAGTGATGTTTAAGTT-3′; R: 5′-CACTCCAAAAAAATCTTAAACTA-3′. The BSP products were confirmed by 2% agarose gel electrophoresis. Finally, the products were cloned into apMD19-T (TaKaRa, Kusatsu, Japan) and sequenced (TsingKe). The sequencing results were analyzed using BiQ Analyser software.

### Differential gene expression and survival analyses

The mRNA and miRNA expression profiles and corresponding clinical data from HCC and non-carcinoma tissues were extracted in TCGA through their portal website for analysis [49]. Expression profiles and tumor types were analyzed using *t* tests. Differences in expression in tumor versus non-carcinoma tissues were analyzed with a threshold of *P* < 0.05. Data analysis was performed using R software (Version 4.0.3). Kaplan–Meier curves were generated to compare survival time differences. *P* values were determined using the log-rank test, with *P* < 0.05 indicating statistical significance.

### Protein interaction analysis

Protein interactions were identified through the STRING database, which is available online at https://string-db.org/.

### Statistical analysis

All results are presented as the mean ± standard deviation. Between-group differences were analyzed using Student’s *t* tests (2-sided), while among-group differences were examined through one-way ANOVA. The representative images or statistical results are based on 3 or more independent experiments. *P < 0.05; **P < 0.01; ***P < 0.001; ns, not significant. These analyses were performed using GraphPad Prism software.

## Data Availability

The data supporting the findings of this study are openly available in its online Supplementary Materials. The raw data related to miRNA-seq underlying this article are available at https://www.ncbi.nlm.nih.gov/bioproject/PRJNA1029726.
