# Supplementary material for: ATPase Inhibitory Factor 1 Drives Mitochondrial Energy Metabolic Reprogramming to Promote HCC Vasculogenic Mimicry via the ESR1/miR-20a-3p/GNAZ Pathway
Source: Research (Wash D C). 2025 Nov 25;8:0998. doi: 10.34133/research.0998 (PMC12645449; doi:10.34133/research.0998)
Supplement: Supplementary 1 — Figs. S1 to S7 Tables S1 to S7 [file research.0998.f1.zip › Supplementary table 7.docx]

**Supplementary table 7**. Antibody of western blot.

| Antibody | Company | Concentrations | Catalog Number |
| --- | --- | --- | --- |
| IF1 | Abcam, London, UK | 1:1000 | Ab223779 |
| VE-cadherin | Abcam, London, UK | 1:1000 | Ab33168 |
| E-cadherin | Abcam, London, UK | 1:1000 | Ab231303 |
| MMP2 | Abcam, London, UK | 1:1000 | Ab92536 |
| MMP9 | Abcam, London, UK | 1:1000 | Ab76003 |
| GNAZ | Abcam, London, UK | 1:1000 | Ab154846 |
| ERK1+ERK2 | Abcam, London, UK | 1:500 | Ab184699 |
| p-ERK1+ERK2 | Abcam, London, UK | 1:500 | Ab201015 |
| c-Fos | Abcam, London, UK | 1:500 | Ab222699 |
| p-c-Fos | Abcam, London, UK | 1:500 | Ab308128 |
| c-Jun | Abcam, London, UK | 1:500 | Ab40766 |
| p-c-Jun | Abcam, London, UK | 1:500 | Ab32385 |
| ESR1 | Abcam, London, UK | 1:1000 | Ab237995 |
| β-actin | ABclonal, Boston, USA | 1:2000 | AC028 |
| GAPDH | Abcam, London, UK | 1:2000 | Ab181602 |
| PDH | Abcam, London, UK | 1:500 | Ab110416 |
| CV-ATP5A | Abcam, London, UK | 1:4000 | Ab110413 |
| CIII-UQCRC2 | Abcam, London, UK | 1:4000 | Ab110413 |
| CIV-MTCO1 | Abcam, London, UK | 1:4000 | Ab110413 |
| CII-NDUFB8 | Abcam, London, UK | 1:4000 | Ab110413 |
| CI-NDUFB8 | Abcam, London, UK | 1:4000 | Ab110413 |
