# Supplementary material for: ATPase Inhibitory Factor 1 Drives Mitochondrial Energy Metabolic Reprogramming to Promote HCC Vasculogenic Mimicry via the ESR1/miR-20a-3p/GNAZ Pathway
Source: Research (Wash D C). 2025 Nov 25;8:0998. doi: 10.34133/research.0998 (PMC12645449; doi:10.34133/research.0998)
Supplement: Supplementary 1 — Figs. S1 to S7 Tables S1 to S7 [file research.0998.f1.zip › Supplementary information.doc]

**Supplementary Information**

**Supplementary Figure legends**

**Supplement Fig. 1.** IF1 promotes HCC VM formation and tumor growth

(A) Image J quantitative analysis showing that IF1 protein expression in IF1-knockdown HCCLM3 cells was reduced compared to the control group. **(B)** Image J quantitative analysis showing that IF1 protein expression in IF1-overexpressing HepG2 cells was increased compared to the control group. **(C)** Image J quantitative analysis showing that after IF1 knockdown, VE-cadherin, MMP2, and MMP9 protein expression in HCCLM3 cells was reduced, while E-cadherin expression was increased. **(D)** Transwell migration assay showing significantly enhanced migration ability of IF1-overexpressing HepG2 cells. **(E)** Tube formation assay showing significantly increased number of tube-like structures formed by IF1-overexpressing HepG2 cells. **(F)** Quantitative analysis showing that VM density in tumor tissues of the IF1-overexpression group was higher than that of the control group. **(G)** Immunofluorescence staining showing decreased E-cadherin expression and increased VE-cadherin expression in IF1-overexpressing HepG2 cells. **(H)** Western blot showing downregulated E-cadherin protein expression and upregulated VE-cadherin, MMP2, and MMP9 protein expression in IF1-overexpressing HepG2 cells. **(I)** Nude mouse subcutaneous xenograft model showing that mice injected with IF1-overexpressing HepG2 cells had significantly larger tumor volume than the control group (n=5/group). **(J)** CD31/PAS staining showing significantly higher VM density in tumor tissues of the IF1-overexpression group than in the control group. **(K)** IHC staining showing upregulated VE-cadherin, MMP2, and MMP9 expression and downregulated E-cadherin expression in tumor tissues of the IF1-overexpression group. Error bars represented mean ± SD. The *t* test was used for statistical analysis (ns, not significant, **p* <0.05, ***p* < 0.01, ****p* <0.001).

**Supplement Fig. 2.** Single-cell sequencing analysis of HCC **(A)** Quality control chart of the GSE166635 single-cell data set. **(B)** tSNE plots for the cell type identification of high-quality single cells from HCC. **(C)** Normalized expression of known genes on a UMAP plot to identify different cell types. **(D)** Heatmap of clustered feature genes for each subpopulation.

**Supplement Fig. 3.** IF1 overexpression inhibits miR-20a-3p expression through promoting ROS accumulation **(A)** Mitochondrial morphology of control and IF1-overexpression HepG2 cells. **(B)** Representative image showing JC-1 fluorescence in control and IF1-overexpression HepG2 cells. **(C)** Intracellular ATP content in control and IF1-overexpression HepG2 cells. **(D)** Western blot showing mitochondrial complexes (I-V) expression levels in control and IF1-knockdown HCCLM3 cells. **(E)** Quantitative analysis of cellular ROS levels in control and IF1-knockdown HCCLM3 cells. **(F)** ROS levels were detected in control and IF1-knockdown HCCLM3 cells with and without the addition of ROS agonist. **(G)** and **(H)** The levels of cellular ROS in control and IF1-overexpression HepG2 cells were assessed. **(I)** Detection of mitochondrial superoxide and cytoplasmic hydrogen peroxide showing significant increase of both in IF1-overexpressing HepG2 cells. **(J)**  DCFH-DA staining showing that IF1 overexpression increased ROS levels in HepG2 cells, and adding a ROS inhibitor reduced ROS levels. **(K)** RT-qPCR showing that IF1 overexpression significantly decreased miR-20a-3p expression, and this effect was reversed by adding a ROS inhibitor. ROS, reactive oxygen species. Error bars represented means ± SD.The *t* test was used for statistical analysis (ns, not significant, **p* <0.05, ***p* < 0.01, ****p* <0.001).

**Supplement Fig. 4.** Methylation analysis of ESR1 **(A)** Prediction of CpG island location in the ESR1 gene promoter region using the UCSC Genome Browser, and design of 5 pairs of bisulfite PCR (BSP) primers (F1-R1 to F5-R5) covering key regions of the CpG island. **(B)** PCR detection for ESR1 gene promoter methylation.Methylation-specific PCR (MSP) results: Lane M is DL2000 Marker, Lanes 1-9 are different HCC cell samples, showing the distribution of ESR1 promoter methylation bands (amplified by methylation-specific primers) and unmethylated bands (amplified by unmethylated-specific primers), confirming the validity of the detection method.

**Supplement Fig. 5.** IF1 promotes HCC cell migration and tube formation by inhibiting miR-20a-3p. **(A)** RT-qPCR confirming that co-transfection with miR-20a-3p mimics significantly increased miR-20a-3p expression in IF1-overexpressing HepG2 cells. **(B)** RT-qPCR confirming that co-transfection with a miR-20a-3p inhibitor significantly decreased miR-20a-3p expression in IF1-knockdown HCCLM3 cells. **(C)** Image J quantitative analysis showing that IF1 knockdown increased E-cadherin and decreased VE-cadherin, MMP2, and MMP9, respectively, in HCCLM3 cells, and co-transfection with a miR-20a-3p inhibitor reversed this trend. **(D)** Transwell migration assay showing that IF1 overexpression significantly enhanced the migration ability of HepG2 cells, and co-transfection with miR-20a-3p mimics reversed this effect. **(E)** Tube formation assay showing that IF1 overexpression significantly increased the number of tube-like structures formed by HepG2 cells, and co-transfection with miR-20a-3p mimics reduced the number of tubes. **(F)** Western blot showing that IF1 overexpression downregulated E-cadherin and upregulated VE-cadherin/MMP2/MMP9 in HepG2 cells, and co-transfection with miR-20a-3p mimics reversed this protein expression pattern. **(G)** Immunofluorescence showing that IF1 overexpression decreased E-cadherin and increased vimentin in HepG2 cells, and co-transfection with miR-20a-3p mimics restored their expression. Error bars represented means ± SD. Statistical analysis was performed using one-way ANOVA (ns: no significant difference, *p<0.05, **p<0.01, ***p<0.001).

**Supplement Fig. 6.** IF1 promotes tumor growth, VM formation and lung metastasis by inhibiting miR-20a-3p. **(A)** Orthotopic HCC xenograft model showing that mice injected with IF1-overexpressing HepG2 cells had significantly heavier tumor weight than the control group, and co-transfection with miR-20a-3p mimics reduced tumor weight (n=6/group). **(B)** Lung metastasis count showing significantly more lung metastatic foci in the IF1-overexpression group than in the control group, and co-transfection with miR-20a-3p mimics reduced the number of metastatic foci. **(C)** CD31/PAS staining showing significantly higher VM density in tumors of the IF1-overexpression group than in the control group, and co-transfection with miR-20a-3p mimics reduced VM density. **(D)** IHC staining showing that the IF1-overexpression group had downregulated E-cadherin and upregulated VE-cadherin/MMP2/MMP9 in tumors, and co-transfection with miR-20a-3p mimics reversed this expression pattern. Error bars represent mean±SD. Statistical analysis was performed using one-way ANOVA (ns: no significant difference, *p<0.05, **p<0.01, ***p<0.001).

**Supplement Fig. 7.** GNAZ is a direct target of miR-20a-3p. **(A)** TCGA database analysis showing significantly higher GNAZ expression in HCC tissues than in normal liver tissues. **(B)** Kaplan-Meier survival analysis showing significantly shortened overall survival in HCC patients with high GNAZ expression. **(C)** Correlation analysis from TCGA database showing positive correlations between GNAZ and CDH2, vimentin, MMP2, and MMP9 expression; **(D)** Western blot showing significantly increased GNAZ protein expression in IF1-overexpressing HepG2 cells. **(E)** Western blot showing significantly decreased GNAZ protein expression in IF1-knockdown HCCLM3 cells. **(F)** Western blot showing significantly decreased GNAZ protein expression in miR-20a-3p-overexpressing HepG2 cells; **(G)** Image J quantitative analysis showing that GNAZ protein expression in HepG2 cells transfected with a GNAZ overexpression plasmid was increased compared to the control group. **(H)** Image J quantitative analysis showing that GNAZ overexpression downregulated E-cadherin and upregulated VE-cadherin, MMP2, and MMP9 in HepG2 cells. **(I)** Tube formation assay showing that GNAZ overexpression significantly increased the number of tube-like structures formed by HepG2 cells, and this effect was reversed by adding an ERK inhibitor. Error bars represent mean ± SD. Statistical analysis was performed using t-test or one-way ANOVA (ns: no significant difference, *p<0.05, **p<0.01, ***p<0.001).
